# A comprehensive assessment of quality management methods in the SMESH study

**DOI:** 10.1186/s12913-024-11055-3

**Published:** 2024-05-20

**Authors:** Bruna Vieira Fernandes, Natália Luiza Kops, Luana Giongo Pedrotti, Tássia Rolim Camargo, Eliana Marcia Wendland

**Affiliations:** 1 Graduation in Biomedicine, Federal University of Health Sciences of Porto Alegre (UFCSPA), Porto Alegre, Brazil; 2 PROADI-SUS, Hospital Moinhos de Vento, Porto Alegre, Rio Grande do Sul Brazil; 3 Department of Public Health, Federal University of Health Sciences of Porto Alegre (UFCSPA), Porto Alegre, Brazil

**Keywords:** Epidemiological methods, Quality Control, Test-retest reliability, Quality Management, HPV

## Abstract

**Background:**

This paper aims to instigate discussion and publication of methodologies applied to enhance quality management through comprehensive scientific reports. It provides a detailed description of the design, implementation, and results of the quality control program employed in the SMESH study.

**Methods:**

Cross-sectional, multicenter, national study designed to assess the prevalence of human papillomavirus in sex workers and in men who have sex with men (MSM). Respondent-driven sampling recruitment was used. An online system was developed for the study and checkpoints were defined for data entry. The system checked the quality of biological samples and performed a retest with part of the sample.

**Results:**

A total of 1.598 participants (442 sex workers and 1.156 MSM) were included. Fifty-four health professionals were trained for face-to-face data collection. The retest showed Kappa values ranging between 0.3030 and 0.7663.

**Conclusion:**

The retest data were mostly classified as indicating a strong association. The data generated by the checkpoints showed the successful implementation of the quality control program.

## Background

Population-based studies play a fundamental role in data collection and systematization, providing valuable insights that assist authorities to guide health public policies [1, 2]. Ensuring studies are conducted with rigorous efforts to minimize bias and producing trustworthy, reproducible data are is crucial [3]. Quality control (QC) tools are essential to controlling those biases [4]. They can be subdivided into quality assurance (QA), which avoids variations and nonconformities in the process, and is part of the planning phase; and QC itself, which allows researchers to identify, measure, analyze, and correct discrepancies throughout the process [5].

Evaluating the importance of observational studies can be challenging, as they are often reported with inadequacies and inconsistencies, making it difficult to assess their reliability and generalizability. These parameters are fundamental, and studies must adhere them to be included in evidence synthesis and systematic reviews that lead to changes in politics and practices [6]. The available instruments for assisting with the critical evaluation of observational studies show a discrepancy in quality compared to those designed for controlled trials [7]. The management of studies with multicultural, multiregional, and multicenter aspects require careful attention to elaborate quality indicators, as described by Harkness et al. [8]. Despite the existence of reference materials and guidelines in the scientific community for conducting studies and publishing results, there is no consensus about essential aspects for QA and QC in different study designs worldwide [7, 9, 10]. Scientific studies often lack a detailed section about the projection, execution, and results of the QC methods employed in the research process.

Therefore, seeking to instigate discussion and publication of methodologies applied to enhance quality management through comprehensive scientific reports, this paper aims to provide a detailed description of the design, implementation, and results of the quality control program employed in the SMESH study. This was a multicentre cross-sectional study designed to assess the national prevalence of human papillomavirus (HPV) and infection types in two key populations in Brazil, from 2019 to 2023 [11]. The respondent-driven sampling (RDS) method was used to recruit participants. SMESH study encompassed from a minimum of two state capitals within every microregion across the country. Primary care units and centres for testing and counselling in each city were selected based on their representativeness of the health districts and their resources for collecting and storing biological samples.

## Methods

The sample was composed of sex workers (men and women) and men who have sex with men (MSM), recruited in the all five regions of Brazil. RDS method combines snowball sampling with weighting. This is a modified form of chain referral sampling which uses dual incentives (incentives for both study participation and peer recruitment) and probability weights to offset non-random recruitment. This is the most appropriate method among available alternatives for the inclusion of large and mostly hidden social networks of sex works and gay men/MSM. On the other hand, RDS weights have the potential to introduce bias if there are inaccuracies in how they are measured or if the assumptions upon which the weights are based are not valid [12].

RDS starts with the identification of the first participants in the study, called “seeds”, who were asked to select other members from their social networks [13]. In this study, the invitation was performed by researchers and social movements for represent different sociodemographic and sexual characteristics within the sex workers and gay men/MSM community. Initially, 3 seeds (initial participants) for each key population were selected per city. The number three was defined to result in long recruitment chains, whereby the final sample is not biased by the initial convenience sample of seeds. All participants received 3 coupons to invite friends and acquaintances who met the inclusion criteria of the study. Thus, the study successively reached more people from those populations. The inclusion criteria were described previously [11].

The study had ethical approval from Hospital Moinhos de Vento (number 2.837.840) and other coparticipant centers. After signing an informed consent, participants answered a questionnaire and provided biological samples from the oral, genital (cervical collection for women and a self-collection penile/scrotal for men), and anal regions. Tests for HIV, syphilis, and hepatitis B and C were also offered. Data were collected in testing and counseling centers and primary health care units by professionals previously trained by researchers from the main study team.

### Quality assurance and control

**Fig. 1:**
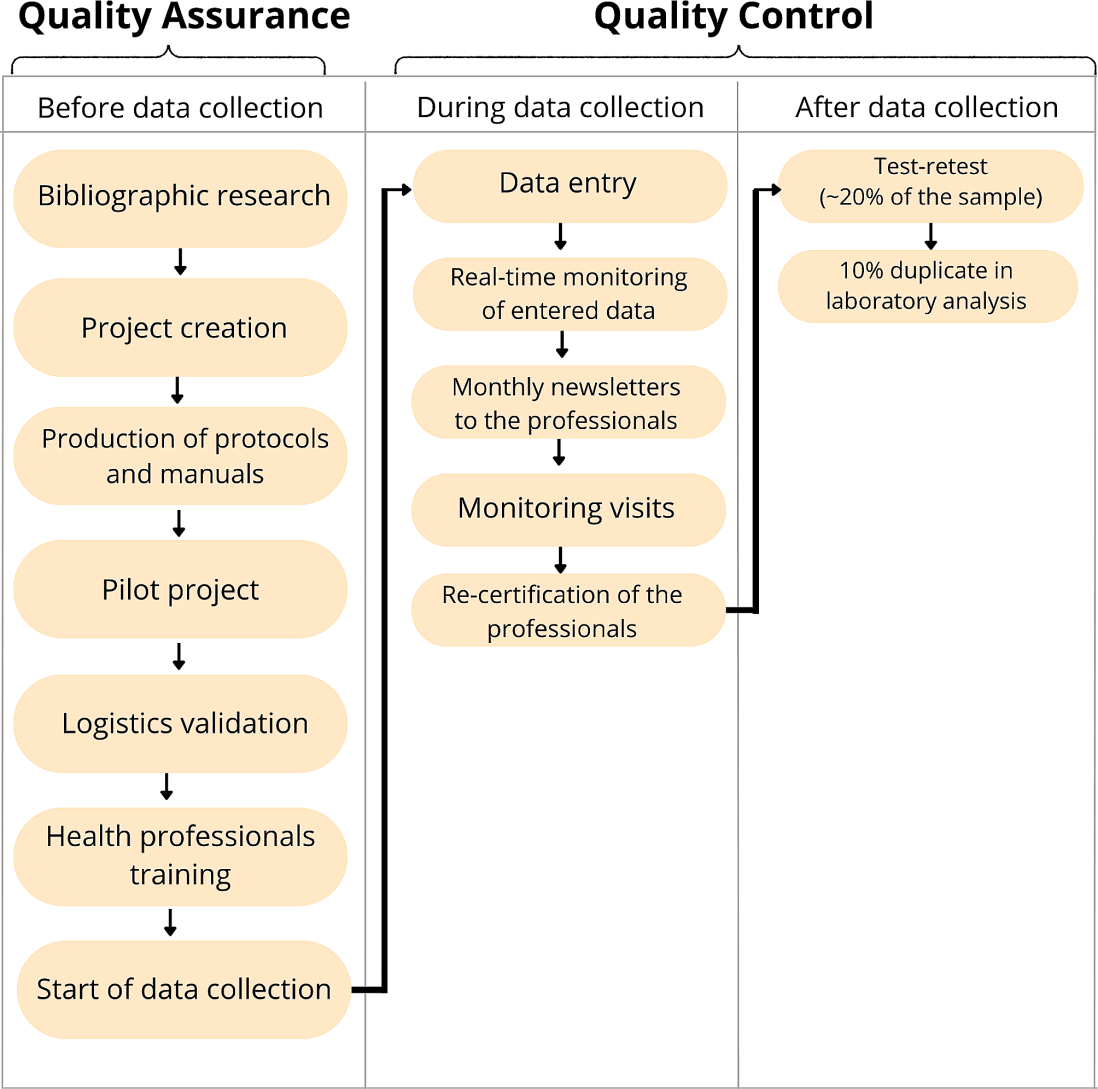
Flowchart with details of quality management measures in each stage of the SMESH study

The methodologies for study quality assurance and control were developed to cover strategic checkpoints. Figure 1 shows items developed in each stage.

Initially, formative research was carried out as part of the RDS method. There were meetings with representatives of social movements from the different research target groups. Representatives collaborated on adjustments to the vocabulary of the questionnaire, the duration of the interviews, indications of possible places to invite participants, and questions about relationship networks maintained between target populations of the study.

An operational manual was created for the professionals trained for data collection. It provided instructions about the entire conduct of the study, including explanations of each question on the questionnaire and a step-by-step commentary about the collection of biological samples. Another manual was created for the researchers who work in the central laboratory used in the study. It contained explicit and objective instructions about the handling of biological samples, from their transport, receipt, and storage to the analysis and publication of the results.

The questionnaire used for data collection was based on previously validated questionnaires. It contained nine sections that grouped questions by area. A pre-test was carried out to certify its functionality, suitability, and feasibility. At the end of the questionnaire, there were free spaces to record several other data points, such as failure in the original data collection protocol, which included participants using mouthwash and gargling for less than 30 s, visible blood in the sample, or intercourse less than 24 h before.

An online data entry platform was developed for the study. Interviews were conducted directly on this platform and, in cases where they were conducted without internet connection, they were synchronized with the central database daily. The system was structured to omit questions that were not applicable (skip questions) and to not allow progress in the instrument in case of non-response or inadequacy in the format. The same system stored analysis and processing data related to the biological samples, which was entered at different moments, from collection to transport and final analysis.

The central laboratory in the study has a proficiency certification issued by World Health Organization to perform HPV genotyping [14]. Team members were trained according to good clinical practices. They were also trained to operate each piece of equipment or system required.

Professionals responsible for data collection from the questionnaire were trained *in loco* by researchers from the main study team. At the end of training, they answered a test with 10 questions and performed a supervised collection.

At the ends of the participant interviews, biological samples were collected from the oral, genital, and anal regions. Samples were sent to the central laboratory within a maximum of 15 days. The samples were stored in a refrigerator or in a climatically controlled environment (air-conditioned room or thermal boxes with gel packs provided by the study)in the health unit and their temperature was monitored using thermometers also provided by the study team. When the temperature was outside the ideal range (2° to 25 º C), the thermometer would trigger an alarm. Consequently, the collector had to adjust the temperature of the location.

Once received at the central laboratory (the Laboratory of Clinical Epidemiology of the Federal University of Health Sciences of Porto Alegre), biological samples first had their temperature analyzed and were then analyzed for their conformity to acceptability criteria, such as volume and adequate identification.

Transport of biological samples was carried out by a company specializing in air logistics. For temperature management during the transport, thermometers were used in random shipments to record variations.

Samples were submitted to DNA extraction, detection, and genotyping tests for HPV. After extraction, nucleic acids were evaluated according to absorbance ratio A260/A280. Samples with a ratio lower than 0.6 were considered inadequate and were not submitted to the genotyping process [15]. Samples with a ratio greater than 0.6 were only considered inadequate after being submitted to 2 previously defined protocols for samples with lower quality.

Backups of both original samples and their purified DNA were stored and cataloged in ultra-freezers. A subset of 10% of the samples was drawn to verify the HPV results by intra-observer, which did not know previous results. The cataloging of the samples was analyzed on the online platform and samples were identified with bar codes.

To ensure reliable analyses without any contamination, ultrapure water was periodically included in all PCR assays as a control reaction.Detection and genotyping for HPV were done using the Anyplex™ II HPV28 Detection test (Seegene), which uses the human β-globin gene as an internal reaction control.

Data entry monitoring was done continuously throughout the study for observation of trends and of errors or incompatibilities in both questionnaire answers and biological samples. From this monitoring, it was possible to prepare newsletters, which were sent to data collectors monthly. Those newsletters had rankings of the greatest data collectors of the month and graphs to illustrate the percentage of goals already achieved. Throughout the study period, the collectors had WhatsApp® groups in which they could exchange messages with the main researchers to resolve doubts and strengthen contacts.

As a result of the COVID-19 pandemic, study activities were suspended for different periods and resumed according to availability of professionals and health standards in each city/state. Before data collection resumed, the health professionals completed an online questionnaire. The questionnaire was developed to assess whether they were still familiarized with the study and able to perform the procedures.

During the study, monitoring visits were conducted at the centers. The main researchers monitored data collection to verify that data met study protocols. Necessary adjustments were requested at the ends of visits.

To perform the retest, we planned to randomly select at least 20% of the sample and a part of the questionnaire was redelivered by telephone call by three researchers from the main team. WhatsApp® messages were sent to schedule suitable days/shifts to contact participants. This questionnaire included questions to ensure that the study procedures were done correctly, in addition to questions about sexual behavior, smoking, health status, education, and participant identification. Participants were also asked about collection of rapid tests for HIV and syphilis. This question was not included in the agreement analysis because it referred to tests during the study participation in the retest stage, and on the other hand, it referred to a previous participant’s life moment in the original questionnaire. Questions related to identification of the participants were not included in the analyses.

Categorical variables were represented by absolute and relative frequencies, while continuous variables were represented by mean and standard deviation. Agreement between study answers and retest answers was evaluated by Cohen’s Kappa (k), which was classified according to criteria proposed by McHugh as follows: 0–0.20 = no agreement; 0.21–0.39 = minimal agreement; 0.40–0.59 = weak agreement; 0.60–0.79 = moderate agreement; 0.80–0.90 = strong agreement; and > 0.90 = almost perfect agreement [16]. Cohen’s Kappa was selected as the specific statistical measure due to its suitability for assessing inter-rater agreement. However, one limitation of Cohen’s Kappa is its sensitivity to the prevalence of agreement in the data. All analyses were performed using SAS (Statistical Analysis System, SAS Institute Inc., Cary, N.C.) version 9.4, and the significance level considered was 5%.

## Results

A total of 56 seeds were selected through the RDS method and, to date, 1598 participants have been included in the study (1191 men and 407 women). The sample included Brazilian and some foreigner participants (Venezuelan, Peruvian and South Korean), which were all residents in Brazil. At first, the question about nationality was not included in the original questionnaire. However, there was an increase in Venezuelan immigration to the North of Brazil, at the beginning of the study, and then it was necessary to include it. As it was added later, there were some missing for this question. The mean age was 30.9 ± 10.4 years, ranging from 18 to 75 years. Around 31.5% of respondents had incomplete higher education and 28.2% had secondary/technical education. As for skin color/race, 41.7% declared themselves brown, 36.1% white, 20.6% black, 0.9% indigenous and 0.7% asian.

In total, 54 health professionals underwent training for the data collection. Training was carried out in 16 primary health care units or testing and counseling centers in 9 Brazilian capitals: Porto Alegre, Florianópolis, Belo Horizonte, Rio de Janeiro, Recife, João Pessoa, Manaus, Boa Vista, and Campo Grande. Data collectors obtained a mean of 9.2 correct answers (of a total of 10) in the post-training test. The questionnaire administered after activity suspension due to the COVID-19 pandemic had 22 respondents, and all of them were approved.

The central laboratory received 215 shipments of samples, with a mean of 2 days in transport and a range of 0 to 8 days. The interval between samples being obtained and results being available to participants via the study system ranged from 2 to 309 days, with a mean of 30 days. Temperature records showed that 56.5% of shipments were kept refrigerated between 2ºC and 8ºC, and 31.6% of shipments ranged from 8ºC to 25ºC. For temperatures up to 25ºC, samples were considered inadequate and then were discarded.

Protocol failures were documented during the collection process as follows: 1.13% of the oral samples, 3.23% of the cervical samples, 0.25% of the penile samples, and 0.38% of the anal samples. Such failures could be due to penile collection performed by the collector instead of self-collection by the participant and bleeding during anal or cervical collection. Laboratory data showed 122 (7.7%) records indicating inadequacy in the received samples. Of the 4780 samples suitable for processement, 56 were inadequate for HPV detection and genotyping. None of the ultrapure water samples presented valid results.

**Fig. 2:**
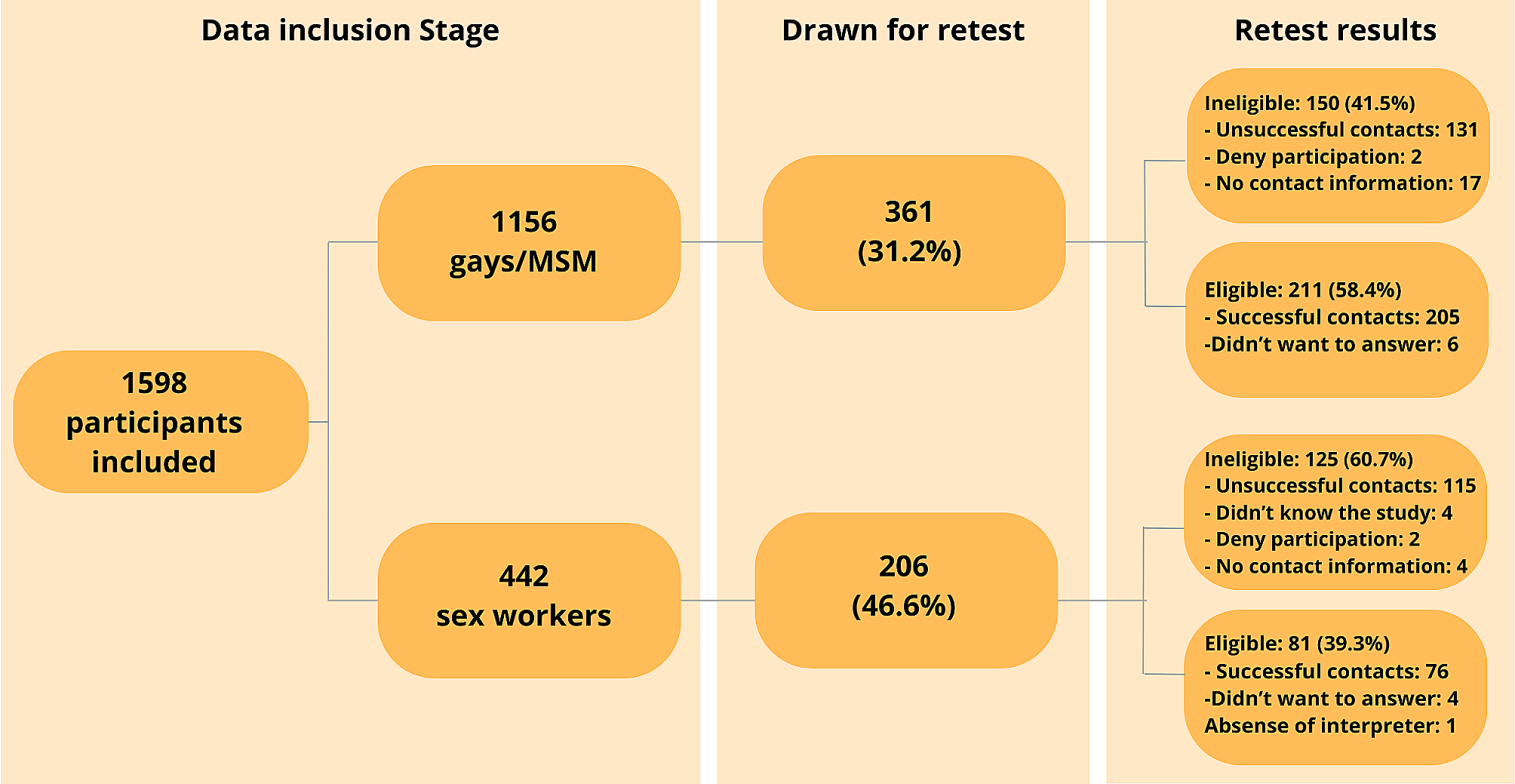
Flowchart with contact attempts in the retest stage

In the retest stage, 567 participants were randomly selected (361 MSM and 206 sex workers — 178 women and 28 men). Of those, 211 (58.4%) and 81 (39,3%), respectively, were contacted successfully (Fig. 2). The median time passed between the first interview and the retest was 30 (20–63).

The retest sample presented the following characteristics: Brazilian and Venezuelan nationality and a mean age of 32 ± 10.9 years (ranging from 18 to 67 years). In terms of education, 36.7% had incomplete higher education, 25.2% had secondary/technical education, and 24.1% had complete higher education. As for skin color/race, 39.6% declared themselves white, 36.7% brown, 18.9% black, 2.6% asian, 1.8% indigenous and 0.4% did not want to answer. Agreement analysis data ranged between 0.7663 and 0.3030 (Table 1). The good agreement among sociodemographic data reflects the reliability of the study, indicating that the participants were indeed interviewed. Lower Kappa values were associated to the questions “In general, how do you rate your health status?” (k = 0.3030) and “Who gave you the coupon to participate in this study?” (k = 0.4446). The participant’s health status may have genuinely changed over time, potentially biasing the response, despite the expectation and orientation in the call that the participant should respond based on their status at the time of the interview.

Although information about rapid tests was not included in the agreement analysis, our participants reported that they had done an HIV rapid test and two reported they had done a syphilis test without really having done them.

**Table 1:** Agreement between the responses of the participants in the first interview and in the retest

Description of the question	Kappa	Classification*
Sexual orientation	0.7663	Moderate
Self-declared skin color/race	0.7338	Moderate
Education	0.6926	Moderate
Gender of the person of first intercourse	0.7710	Moderate
Smoking of 100 cigarettes	0.6972	Moderate
Who gave the coupon to participate in the study	0.4446	Weak
Rate of health status	0.3030	Minimal

* Classification according to criteria proposed by McHugh, 2012

## Discussion

Successful implementation of quality control processes ensures that collected data is reliable, in addition to generating improvements in processes considered critical. The SMESH study implemented checkpoints to monitor data entry, sample logistics, the knowledge level of professionals, and statistical tests to ensure the reliability of the answers. The training of the research team, as well as the monitoring visits and newsletters, ensured that all people involved in the study were aware of their role in providing good-quality data [17].

The combination of in-person training sessions conducted by researchers from the main study team and online meetings produced a quality culture among the professionals engaged in the research. Their collaboration in implementing essential tools for quality control contributed to the success of the study.

In the recruitment method used (RDS), the participants are responsible for recruiting other individuals [18]. This method relies on sequential recruitment waves, which in the SMESH study were paused due to the COVID-19 pandemic. When study activity resumed, telephone calls for the retest stage were also a strategy to restart coupon distribution. A questionnaire was administered and, at the end of the call, the researchers encouraged participants to invite their peers to continue waves of recruitment. For this reason, for the retest we randomly selected more sex workers because they had the lowest recruitment rate at the time of analysis.

In addition to impacting participant recruitment and logistics, the pandemic certainly influenced participants’ responses. For example, in access to condom use, frequency of health facility visits, and testing for STIs.

The SMESH study has a wide geographical dispersion. Therefore, due to the importance of maintaining sample quality, logistic processes required constant monitoring to ensure that samples were not affected during transportation. Records showed that one third of the shipments were received with temperatures higher than the expected (up to 8 °C); however, they were still within the safety margin (25ºC). The period between sample collection and availability of the genotyping results to the participants was 30 days, as planned, and intervals higher than 60 days were identified only during the COVID-19 pandemic.

Samples that did not obtain valid results accounted for 0.85% of collected data. This information, as well as no amplification found in the ultrapure water samples submitted to the processes, shows the quality of research team members and the importance of using automated analytics. These actions generated samples that were without contamination and were reproducible [19].

Analysis of the questionnaire administered for the retest showed moderate agreement in Kappa values for 5 of 7 questions, which ensured that answer agreement was not random. Values that indicated no agreement and minimal agreement, respectively, possibly due to misunderstanding or confusion about the questions, as they referred to the moment of participation in the study and not to the moment of the telephone call. As one of these questions pertains to the participant’s health status, it is highly likely that the pandemic may have influenced this perception. Even so, no adjustments were made to this question when analyzing the final data of the study.

Despite the relevance of the study, there are limitations. Duo to logistical and financial reasons, not all capital cities were included. Additionally, the sampling process, particularly in selecting the “seeds”, may introduce bias and fail to represent the entire population.

## Conclusion

The current manuscript shows the various methods employed for quality control in this multicentric, national study. The quality assurance process is expected to produce research that can generate higher-quality data. By incorporating audit points throughout the study, the integrity and reliability of the research are ensured, leading to more robust and credible results.

## Data Availability

The datasets used and/or analysed during the current study are available from the corresponding author on reasonable request.

## Abbreviations

MSM: Men Who Have Sex With Men
QC: Quality Control
QA: Quality Assurance
SMESH: Sex Workers And Men Who Have Sex With Men: Evaluation Of Sexual Health Study
HPV: Human Papillomavirus
RDS: Respondent-Driven Sampling
